# Prognostic and therapeutic implications of *TP53* expression in chronic myelomonocytic leukemia

**DOI:** 10.1038/s41408-024-01087-7

**Published:** 2024-07-12

**Authors:** Yu-Hung Wang, Chien-Chin Lin, Kristian Gurashi, Chi-Yuan Yao, Andres Jerez, Hsin-An Hou, Wen-Chien Chou, Hwei-Fang Tien, Kiran Batta, Daniel H. Wiseman

**Affiliations:** 1 Division of Cancer Sciences, Epigenetics of Haematopoiesis Laboratory, The University of Manchester, Manchester, UK; 2 Division of Hematology, National Taiwan University Hospital, Taipei, Taiwan; 3 Department of Laboratory Medicine, National Taiwan University Hospital, Taipei, Taiwan; 4 Haematology Department, Hospital Morales Meseguer, Murcia, Spain; 5 Department of Internal Medicine, Far-Eastern Memorial Hospital, New Taipei City, Taiwan

**Keywords:** Myelodysplastic syndrome, Genetics research

Dear Editor,

The tumor suppressor *TP53* is the most frequently mutated gene in cancer [1]. Across diverse myeloid malignancies TP53 disruption is common and associated with poor-risk disease [2] and therapeutic resistance [3]. Unusually, *TP53* mutations are rare in chronic myelomonocytic leukemia (CMML) [4, 5], despite disease features otherwise overlapping with myelodysplastic syndromes (MDS), myeloproliferative neoplasms and acute myeloid leukemia (AML): each of which harbors sizeable proportions of *TP53*-mutated cases. Recently, the largest cohort to date reported *TP53* mutations in only 2.4% of 1315 CMML cases [4]. Predictably these patients displayed adverse features and inferior AML-free survival (LFS) and overall survival (OS) compared with *TP53* wild-type (*TP53*^WT^) CMMLs.

While the clinical characteristics of *TP53*-mutated CMML are now comprehensively described [4], this represents a small fraction of CMML cases. Additionally, p53 activity can be modulated by non-mutational mechanisms, for example via altered transcriptional expression, posttranslational modifications, and cellular localization [6]. We hypothesized that p53 dysfunction might otherwise characterize a hitherto-unknown subset of *TP53*^WT^ CMML patients, and so investigated *TP53* mutations, allelic status, expression level, and therapeutic response in a large international collaborative CMML cohort.

We studied 648 CMML patients from North−West England with available clinical, mutational, and outcome data. Subsets of patients treated at The Christie (Manchester, UK) underwent RNA-sequencing on bone marrow (BM) CD34-sorted hematopoietic stem/progenitor cells (HSPCs; *n* = 33); and p53 immunohistochemical (IHC) staining on archived BM trephine samples (*n* = 31; *n* = 14 overlapping both cohorts). Separately, we analyzed 92 patients treated at National Taiwan University Hospital (NTUH, Taipei, Taiwan) for whom presentation BM mononuclear cells (MNCs; *n* = 92) and RNA-sequencing (*n* = 90/92) data were available [7]. Finally, we re-analyzed published RNA-sequencing data from BM MNCs of 24 patients from Hospital Morales Meseguer (Murcia, Spain) [8]. Each cohort included healthy BM controls (HCs). Additional cohort details and experimental methods are provided as Supplementary Data (Methods S1−8, Tables S1−3).

Median ages of the 648 UK and 92 Taiwanese patients were 75 and 71 years, respectively, both with male predominance (Tables S4−5). Only eight (1.23%) and two (2.2%), respectively, carried *TP53* alterations (Fig. 1A, B). As expected, UK *TP53*-altered patients displayed significantly inferior outcomes compared with *TP53*^WT^ (Fig. 1A), with a similar trend in the Taiwan cohort (Fig. 1B). Thus, we corroborate the paucity of *TP53* mutations in CMML [4] across previously unreported UK and Taiwan cohorts.

**Fig. 1:**
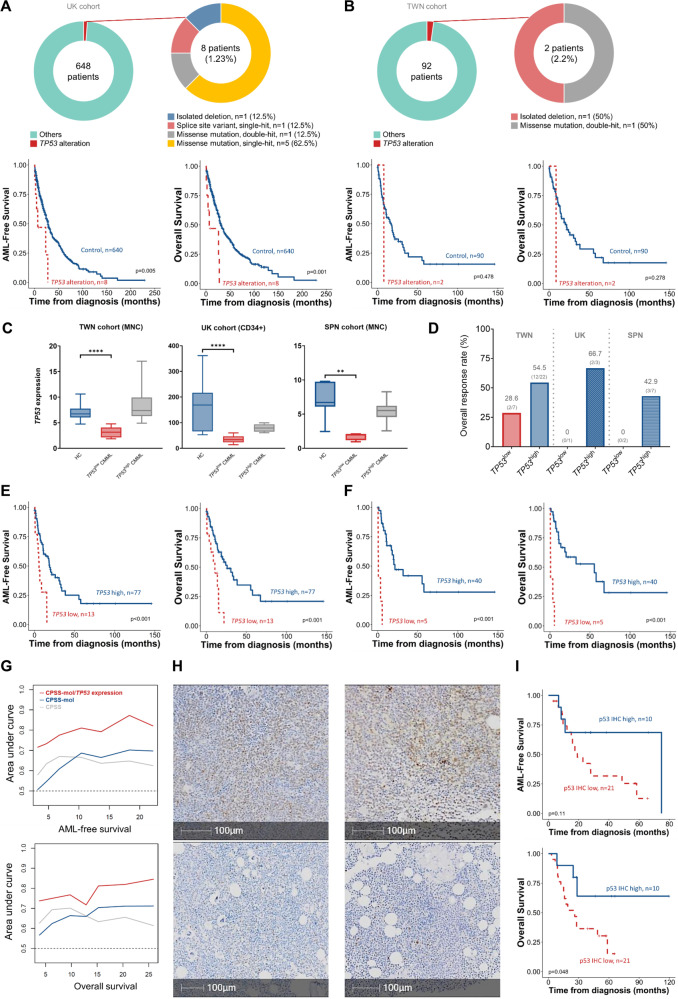
Incidence and prognostic impact of *TP53* alterations and the prognostic implication of *TP53* expression in CMML. **A** Incidence of *TP53* alterations in the UK CMML cohort (upper) and the outcomes of patients with or without any type of *TP53* alteration (lower). **B** Incidence of *TP53* alterations in the Taiwanese CMML cohort (upper) and the outcomes of patients with or without any type of *TP53* alteration (lower). **C** Box and whisker plots displaying *TP53* expression of healthy controls (HC) and CMML patients without *TP53* alterations across three cohorts. MNC: mononuclear cells. ****P ≤ 0.0001, **P ≤ 0.01. *P* values were computed using the Mann–Whitney test. Segregation of patients into *TP53*^low^ and *TP53*^high^ subgroups was performed by the maximally selected rank method. **D** Bar plots showing overall response rates to hypomethylating agent monotherapy in patients with lower and higher *TP53* expression. Numbers in brackets denote responders over the total number of individuals in each group. **E** Low *TP53* RNA expression conferred significantly worse acute myeloid leukemia-free survival (LFS) and overall survival (OS) in CMML patients in the Taiwan RNA-sequencing discovery cohort. **F** *TP53* expression significantly discriminated patients’ LFS and OS in the *ASXL1* wild-type population in the discovery cohort. **G** Time-dependent ROC curve analyses demonstrate that *TP53* expression can refine and improve current prognostic systems. **H** Representative bone marrow sections stained by immunohistochemistry (IHC) for p53 expression from CMML patients in the UK cohort. Nuclei with clear brown color regardless of staining intensity were regarded as p53 positive. Two exemplar high (upper row) and two low (lower row) expressors are shown. **I** Patients with lower p53 IHC expression displayed inferior survival compared to those with higher expression. The cutoff for p53 protein expression (25.4%) distinguishing lower and higher p53 groups was determined using maximally selected rank statistics.

Examining *TP53* gene expression levels across different cell types in normal and malignant hematopoiesis (from publicly available datasets) revealed that healthy HSCs express significantly higher *TP53* than in MDS (*p* < 0.01, Fig. S1). Therefore, we explored *TP53* expression levels and their clinical significance in *TP53*^WT^ CMML cases, similarly observing lower *TP53* in the UK and Spain cohorts (Fig. S2A). Notably, *TP53* expression in HSPCs was significantly higher than in MNCs from both healthy and disease contexts (Fig. S2B).

Our Taiwan RNA-sequencing CMML discovery cohort was stratified into *TP53*^high^ and *TP53*^low^ transcriptional expression groups. *TP53*^low^ patients displayed significantly lower expression than HCs, whereas *TP53*^high^ expression levels were comparable to controls (Fig. 1C). Thus, while most CMML patients are *TP53*^WT^, BM cells from a subset (~15%) display abnormally low *TP53* expression, suggesting potential for altered p53 (and downstream) function in these patients. Considering the functional crosstalk between the MDM2-MDMX complex and p53 we examined correlations between *MDMX*, *MDM2*, and *TP53*. *TP53*^low^ expressors exhibited higher *MDMX* than *TP53*^high^, with no difference observed for *MDM2* (Fig. S3). Clinical and mutational features did not differ between the two *TP53* expression subgroups (Tables S6−11).

Given the association between *TP53* mutations and HMA resistance [3], we examined whether *TP53* expression correlated with HMA response in the *TP53*^WT^ context. Despite limited sample sizes (Table S12), *TP53*^low^ patients showed a consistent trend towards poorer HMA response rates across cohorts (Fig. 1D). *TP53*^low^ patients displayed significantly shorter LFS and OS than *TP53*^high^ cases (Fig. 1E). In subgroup analyses, lower *TP53* retained strong predictive value for LFS and OS even within CPSS and CPSS-Molecular-stratified subgroups (Figs. S4, 5), and in exclusively *ASXL1*^WT^ patients (Fig. 1F). Time-dependent ROC curve analysis revealed potential for *TP53* expression to enhance current prognostication systems (Fig. 1G). In multivariable analysis, lower *TP53* expression remained prognostically detrimental for LFS and OS (Table S13). This was consistent across validation cohorts (Figs. S6, 7; Tables S14, 15), strengthening the observed link between lower *TP53* expression and adverse outcomes.

We subsequently explored TP53 expression at the protein level by IHC in 31 CMML trephine samples (Fig. 1H). We found no significant correlation between p53 IHC and *TP53* RNA-sequencing expression levels: albeit with only a small overlapping cohort with both available (Table S16, 17, Fig. S8), and comparing different populations (whole BM vs CD34 +, respectively). In the full IHC cohort, however, low p53 expressing cases exhibited significantly inferior OS (*p* = 0.048; Fig. 1I), validating our observations comparing *TP53* transcript levels.

We next sought how lower *TP53* expression might influence CMML biology and prognosis. Concordant with our clinical observation (Fig. 1D), single-sample GSEA showed enrichment of HMA resistance signatures in *TP53*^low^ patients, consistently across cohorts (Figs. 2A, S9). We hypothesized that altered *TP53* expression might be associated with aberrant self-renewal and cell cycle programs: recognized mediators of established HMA resistance mechanisms [9]. *TP53*^low^ cells showed enrichment for LSC and HSC genes, and relative decrease in cell cycle-related genes, as compared with *TP53*^high^ and HC (Figs. 2B, S10). Thus, *TP53*^low^ CMML displays distinct stemness and quiescence signatures, linked to poor HMA response in these patients.

**Fig. 2:**
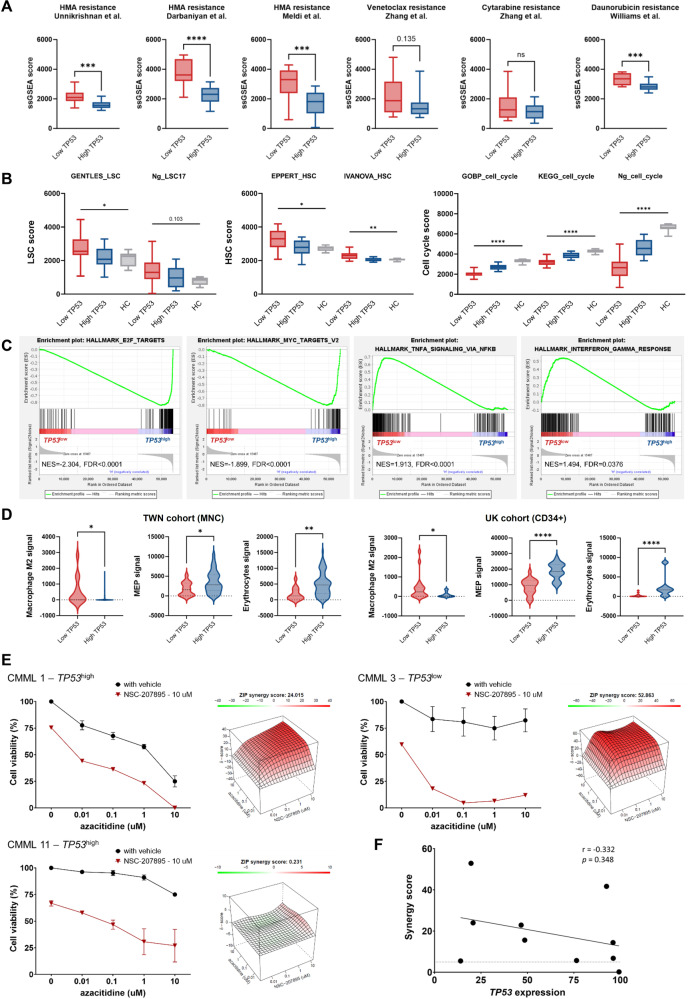
Biological and therapeutic implications of *TP53* expression in CMML. **A** Box plots displaying resistance signatures derived from single-sample GSEA for hypomethylating agents (HMA), venetoclax, cytarabine, and daunorubicin in patients with lower and higher *TP53* expression in the UK CD34 + -sorted cohort. **B** Box plots displaying scores of leukemic stem cell (LSC), hematopoietic stem cell (HSC), and cell cycle of patients with *TP53*^high^ and *TP53*^low^ expression and healthy controls (HC) in the UK CD34 + -sorted cohort. **C** Representative GSEA plots of pathway enrichment in CMML patients with the lowest 25% vs highest 25% *TP53* expression in the UK CD34 + -sorted cohort. **D** Violin plots displaying different signatures seen in the Taiwan discovery cohort and the UK validation cohort. MEP: megakaryocytic-erythroid progenitors. **A**, **B**, **D** ****P ≤ 0.0001, ***P ≤ 0.001, **P ≤ 0.01, *P ≤ 0.05. *P* values were computed using Mann–Whitney or Kruskal–Wallis test. **E** Representative 3D synergy plots using zero interaction (ZIP) model (right) and dose response curves (left) for CMML bone marrow mononuclear cells (*n* = 3 patients; mean + SEM) treated for 72 h ex vivo with NSC-207895 and azacitidine combination at various concentrations. The presence of synergy was determined utilizing the SynergyFinder computational package and the ZIP synergy index where red denotes synergism and green denotes antagonism. A positive synergy score is the percent more cell death than expected. **F** Dot plot displaying the correlation between *TP53* expression and synergy score of 10 patient samples.

By GSEA *TP53*^low^ cells exhibited depleted expression of p53-dependent pathways, including MYC targets, G2/M checkpoints, and DNA repair (Figs. 2C, S11). Interestingly, these were all among the most upregulated pathways in *TP53*-mutant (vs *TP53*^WT^) samples across multiple cancers in TCGA data [1], implying that the driving biology of *TP53*^low^ CMML is distinct from (and not functionally equivalent to) that of oncogenic *TP53* mutations. Conversely, *TP53*^low^ patients demonstrated enhanced TNF-alpha and inflammatory signals (Figs. 2C, S11), highlighting possible crosstalk between p53 and extrinsic factors in the CMML BM microenvironment. Similar results were observed in our other cohorts (Fig. S11). Taken together, compared with HCs or *TP53*^high^ CMML, *TP53*^low^ CMML cells display relatively quiescent cell cycle but heightened inflammation.

With emerging evidence suggesting discrete roles for p53 in regulating inflammation and immune cell landscape [10], we applied xCell to our transcriptomic datasets to analyze signals of 22 cell types. *TP53*^low^ CMML displayed significantly stronger M2-macrophage, but lower megakaryocytic-erythroid progenitors (MEP) signals compared with *TP53*^high^ (Fig. 2D, Table S18). Interestingly, these findings were consistent in the UK CD34+ dataset, suggesting lineage priming at the progenitor level.

Finally, we explored whether reduced *TP53* expression in this CMML subset could be exploited therapeutically. HMAs are the only approved disease-modifying drugs for CMML but often yield disappointing responses [11]. Despite extensive efforts, no combination has yet reported survival advantage over HMA monotherapy. Since the MDM2/MDMX complex degrades wild-type p53, dual inhibition may offer more comprehensive modulation, as suggested by early clinical results in *TP53*^WT^ AML/MDS following HMA failure [12]. Combining NSC-207895, a dual MDMX/MDM2 inhibitor, and p53 activator, with azacitidine at various concentrations, we observed clear and substantial synergy in primary samples ex vivo from 10/11 patients (Fig. 2E; Table S19). There was a trend towards inverse correlation between *TP53* expression and empirical synergy scores (Figs. 2F, S12), suggesting potential for pharmacological p53 activation to enhance HMA sensitivity in CMML with broad efficacy; perhaps preferentially in adverse *TP53*^low^ expressing cases (although we could not validate this experimentally, lacking availability of matched post-treatment samples).

An intriguing question remains: why are *TP53* mutations so infrequent in CMML? Speculatively, *TP53* mutations might induce unknown synthetic lethalities in CMML cells; or they may promote alternative lineage specification pathways, re-directing the expressed phenotype and resultant disease classification. Supporting the latter, most studied *TP53*^MUT^ hematopoietic models report enhanced stemness or propagation of megakaryocytic/erythroid lineage [13], rather than the myelomonocytic expansions that define the CMML phenotype. For example, *TP53* knockout synergized with *NRAS*^G12D^ to specifically transform MEPs, but not other HSPC types, in an AML murine model [14]. Accordingly, we observed significant under-representation of myelomonocytic/blastic M4/M5 FAB subtypes associated with *TP53* mutations amongst 1511 AML cases at NTUH, and re-analyzing 577 cases from TCGA and BeatAML datasets (odds ratio 0.48 and 0.49, respectively; Table S20). Thus, acquisition of *TP53* mutations onto the canonical CMML mutation background might alter the resultant phenotype away from clinicopathological features compatible with CMML diagnostic criteria.

When present, *TP53* mutations confer adverse prognosis in CMML as in other cancers. However, our study identifies prognostic implications of mutation-independent TP53 dysregulation in CMML relevant to a much larger minority of patients (~15%). Prior TCGA analysis revealed substantial variation in *TP53* expression in both *TP53*^MUT^ and *TP53*^WT^ tumors [15], with *TP53*^WT^ expression lower than in missense but higher than in truncating mutations. Furthermore, the relationship between expression and prognosis differed across cancers [15]. Our data suggest that in CMML *TP53* expression level plays a role in dictating disease aggressiveness and therapeutic response, of relatively greater importance than *TP53* mutation status in this disease.

In conclusion, ours is the first study to link low *TP53* expression with distinct features and outcomes in CMML. We confirm the rarity of *TP53* mutations, whilst identifying a novel subgroup with aberrantly low *TP53* expression, associated with higher HMA resistance, distinctive biology, and inferior prognosis. We highlight potential for combining HMA and MDMX/MDM2 inhibition to restore HMA sensitivity, as an attractive candidate therapeutic approach for clinical study to address this unmet clinical need.

### Supplementary information

Supplementary material

Supplementary tables

## Data Availability

The data reported in this article will be provided to collaborating investigators through reasonable request to the corresponding authors after requisite institutional review board approval. The data are not publicly available due to privacy or ethical restrictions.
