# Supplementary material for: Prognostic and therapeutic implications of TP53 expression in chronic myelomonocytic leukemia
Source: Blood Cancer J. 2024 Jul 12;14(1):112. doi: 10.1038/s41408-024-01087-7 (PMC11245466; doi:10.1038/s41408-024-01087-7)

Prognostic and therapeutic implications of *TP53* expression in chronic myelomonocytic leukemia

Supplementary data

Supplementary methods: 1-8

Supplementary figures: 1-12

Supplementary tables: 1-20. Please refer to the supplementary Excel file.

Supplementary Method 1 - Patient cohorts

Our UK cohort comprised 648 consecutive CMML patients diagnosed and treated across North West England between 2009–2023, for whom extensive clinical, molecular and outcome data were available. Full karyotyping data were available for 600 out of 648 patients, some having declining bone marrow (BM) examination or failed metaphase preparation. A subset of 33 patients treated at The Christie NHS Foundation Trust (Manchester, UK) and consented to the Manchester Cancer Research Centre (MCRC) Tissue Biobank underwent RNA-sequencing on BM CD34+ enriched hematopoietic stem and progenitor cells (HSPCs) from cryopreserved mononuclear cell (MNC) preparations. p53 immunohistochemical (IHC) staining on archived BM trephine samples were available for 31 patients, with 14 cases overlapping with the RNA-sequencing cohort. BM samples from seven healthy age-matched individuals, obtained from hip arthroplasty surgeries at Trafford General Hospital (Manchester, UK) in consenting patients, served as healthy controls (HCs).

Separately, we included 92 CMML patients treated at the National Taiwan University Hospital (NTUH; Taipei, Taiwan) for whom cytogenetic data and BM MNC samples at diagnosis were available(1). RNA sequencing data were available for BM MNCs from 90/92 patients. BM MNC samples from 19 healthy hematopoietic stem cell transplant (HSCT) donors were used as HCs. Finally, we re-analyzed and incorporated previously published RNA sequencing data from BM MNCs on 24 CMML patients and seven healthy donors from Hospital Morales Meseguer (Murcia, Spain) as an additional validation cohort(2).

All CMML diagnoses were according to criteria of the 2022 World Health Organization classification(3). Treatment responses were categorized according to the International consortium proposal of uniform response criteria for myelodysplastic/myeloproliferative neoplasms (MDS/MPN)(4). The study was approved by the institutional review boards of each participating hospital, with informed consent obtained throughout in accordance with the Declaration of Helsinki.

Supplementary Method 2 - Immunohistochemical staining for p53 and evaluation

Immunohistochemistry staining was performed using the BOND RX automated platform (Leica Microsystems). 4um sections of FFPE sections were cut and mounted on charged slides. Dewaxing and heat induced epitope retrieval (HIER) of slides was automated on the Bond RX using epitope solution 1 (AR9961) for 20 minutes at 98°C. Using the Bond Refine Kit (DS98007) as per manufacturer's instructions, briefly endogenous peroxidase was blocked (hydrogen peroxide, 10 minutes) followed by a non-specific protein block (casein, 20 minutes) prior to P53 (Dako M7001) (30 minutes) being applied at concentration of 1.185μg/ml. Following buffer washes, the post primary linker was applied for 15mins and the labelled polymer was applied (8 minutes) followed by visualization with diaminobenzidine (10 minutes) and nuclei hematoxylin counterstain (5 minutes). A negative isotype control (mouse IgG2b ADI 20102-103) was applied on several of the samples at a matched concentration. A positive control colorectal carcinoma tissue was also included.

Whole slide imaging was performed using the Olympus VS200 MTL (Olympus Tokyo, Japan), with an Olympus UPLXAPO20X (NA 0.6): 0.274 μm/pixel objective lens. Focus points were chosen to permit a well-defined image across the whole tissue, using intervention to move focus points to any problematic areas. The data were captured on multiple axial planes, then combined using a depth of focus algorithm to maintain focus and render a three dimensional image into two dimensions. Image capture was via an Orca-Flash4 (Hamamatsu Photonics, Germany). Each field of view was captured at 2048x2048 pixels, all under control

from Olympus ASW software. Data were saved directly in the Olympus.VSI format, thus maintaining image capture metadata to a read-only server. Slides were analyzed in Halo 3.6.4134.314 (Indica Labs, New Mexico USA), with manual annotation for tissue region followed by a Random Forest machine learning classifier applied to detect cellular areas. Cell counting was performed using the Multiplex IHC v3.4.9 module with Nuclei detected by DAPI staining with a secondary weighting for DAB staining. Intensity of p53 staining within the nuclear region was then used to define cell positivity. Percentages of p53-positive nuclei were utilized for subsequent analysis.

Supplementary Method 3 - Targeted Mutation Profiling of the UK and Taiwan cohort

Mutation profiling was done as previously described(5, 6). Gene mutations were examined via targeted next-generation sequencing, using the TruSight myeloid sequencing panel (Illumina, San Diego, CA, USA), which included 15 full exon genes and 39 oncogenic hotspot genes. HiSeq platform (Illumina, San Diego, CA, USA) was used for sequencing.

Supplementary Method 4 - Library preparation, RNA sequencing, and analysis pipeline in the UK cohort

BM samples of 33 patients and seven healthy donors were submitted for RNA-sequencing. Extracted RNA was quantified with Qubit and BioAnalyzer as QC step. Samples with high RIN were processed using the NEB Next Ultra II (pA) RNA (New England Biolabs, #E7770) kit following a 10ng input workflow per manufacturer instructions. Samples' libraries were sequenced on a NovaSeq 6000 (Illumina) to obtain a mean of 30M reads per sample and sequenced data demultiplexed using bcl2fastq per manufacturer instructions (Illumina). The sequenced pair-ended libraries were processed using the STAR + Salmon approach (also known as Salmon alignment method). Briefly, fastqs were analysed for quality control using FastQC (v0.11.7) + MultiQC (v1.10.1). Reports did not highlight the need to trim reads, so this was not done. Reads from the representative fastqs were mapped using STAR (v2.5.1b) onto the Gencode v42 genome (with parameter 'quantMode' defined as TranscriptomeSAM and parameter 'twopassMode' as Basic to increase detection power for alternative splicing events). FastQC + MultiQC was re-rerun to verify the quality of the alignment. Then Salmon (v1.4.0) was run using the aligned reads as input to quantify gene reads onto transcripts from Gencode v42 genome, using the parameters 'gcBias' and 'seqBias'. Gene-level summarization was performed using the tximport function from TxImport (v1.26.1) from the quantified transcripts, with countsFromAbundance = 'no'. Non-informative genes with low expression were filtered out (<5 counts in at least 3 samples).

Differential gene expression was performed with DESeq2 (v1.38.3), with fitType = “local” as it presented lower median absolute residuals (better fit). Counts were normalised using the variance stabilizing transformation (VST) to compare gene expression across samples. Significance for the differentially expressed genes across compared groups was corrected using filterFun = ihw (Independent Hypothesis Weighting) (v1.26.0), which using multiple tests leads increased detection power in comparison with the default Benjamini and Hochberg correction. Log fold change was shrinked using the “ashr” setting for visualization and gene ranking (for pathway analyses), and FDR was set at 5%.

Supplementary Method 5 - Library preparation, RNA sequencing, and analysis pipeline in the Taiwan cohort

RNA was extracted from fresh BM samples and sent for RNA sequencing. The TruSeq Stranded mRNA Library Prep Kit (Illumina, San Diego, CA, USA) was used for library preparation, as previously described(7). Briefly, first-strand cDNA was synthesized using reverse transcriptase and random primers. After the generation of double-strand cDNA and adenylation on the 3' ends of DNA fragments, adaptors were ligated and then purified with the AMPure XP system (Beckman Coulter, Beverly, USA). Library quality was assessed using an Agilent Bioanalyzer 2100 system and a real-time polymerase chain reaction (PCR) system. Libraries were sequenced (Genomics, BioSci & Tech Co., Taiwan) on an Illumina NovaSeq 6000 platform with 150 bp paired-end reads generated by Genomics, BioSci & Tech Co., New Taipei City, Taiwan. Low quality bases and adapter sequences in the raw data were removed using Cutadapt (v 3.0)(8). Reads were then aligned to the human genome (GRCh38) and gene annotated with its corresponding GTF files (GENCODE GRCh38) using STAR version 2.4.2a with the settings –outFilterMultimapNmax 20 –outFilterType BySJout –alignSJoverhangMin 8 –quantMode GeneCounts(9). DESeq2 was used to perform differential gene expression analysis and to calculate TPM (transcripts per kilobase million per mapped reads) values for each gene, counting only reads that mapped to exonic regions(10). Genes were called as differentially expressed if they exhibited a Benjamini and Hochberg–adjusted P value (FDR) below 0.05 and a mean fold change of above 2.

Supplementary Method 6 – Bioinformatics analysis

The single sample Gene Set Enrichment Analysis (ssGSEA) algorithm was implemented via the gene-set variation analysis (GSVR) R package, to aggregate the expression values of a gene-set into a single score. Each ssGSEA score reflects the extent to which genes within a specific gene-set are collectively upregulated or downregulated within a single sample for drug resistance profile towards hypomethylating agents (HMAs, derived from three independent studies)(11-13), venetoclax(14), cytarabine(15) and daunorubicin (16). Gene expression signature scores, including those for leukemic stem cell (LSC)(17, 18), hematopoietic stem cells (HSC)(19, 20), and cell cycle(21, 22) signatures, were retrieved from various gene sets described in the literatures (Table S2). Pathway enrichment analysis was conducted using the GSEA application (4.3.2). T-statistics from the differential expression analysis were used to rank genes. xCell, a bioinformatic deconvolution method developed for enumerating distinct cell type signatures from within complex gene expression mixtures, was employed to calculate the enrichment scores for 22 cell types (Table S3)(23).

Supplementary Method 7 – *Ex vivo* drug treatment

BM samples were obtained from the MCRC Tissue Biobank. Samples were donated, with informed consent, from CMML patients at the Christie NHS Foundation Trust (Manchester, UK). BM MNCs were cultured in serum-free medium for expansion and culture of hematopoietic cells (SFEM) supplemented with heat-inactivated (HI) horse serum, 12.5% HI FBS, 1% Penicillin-Streptomycin, 1% 200mM L-glutamine, 1 uM hydrocortisone and 57.2 uM beta-mercapotoethanol. Human cytokines IL-3, TPO, and G-CSF were also freshly added at a final concentration each of 20 ng/ml. Cells were co-cultured with MS-5 stromal cells for 48-72 hours before plating in 96 well plates at 2,000 cells/well and exposed to DMSO vehicle, or azacitidine (Stratech Scientific, S1782-SEL-50mg) and NSC-207895 (Selleckchem, XI-006-5mg), an MDM2/MDMX inhibitor with p53 activating properties, at various concentration ranges with a minimum of three technical replicates per concentration per patient. After 72 hours, 10μl alamarBlue was added to the cells and incubated for 4h at 37°C. Resorufin fluorescence was measured using a fluorescence-based plate reader (POLARstar Omega, BMG Labtech, Aylesbury, UK). Absolute viability values were converted to percentage viability versus control treatment.

Supplementary Method 8 - Statistical analyses

Categorical or nominal variables were compared using the $\chi 2$ or Fisher's exact test. Continuous variables were compared using Mann–Whitney or Kruskal–Wallis tests where applicable. The maximally selected rank statistics implemented in the maxstat R package were used to separate patient into lower and higher expression groups. Maximally selected rank statistics is a statistical method used to determine an optimal cut-off for a continuous variable that best separates groups based on an outcome variable. Survival analyses were evaluated by the Kaplan-Meier method, and curves were compared by log-rank test. The Cox proportional hazard model was used for multivariable analyses. All analyses and visualization were conducted using IBM SPSS Statistics 23 for Windows, GraphPad Prism (10.0.2), and R software (version 4.3.1).

Reference

1. Wang Y-H, Yao C-Y, Lin C-C, Gurashi K, Amaral FMR, Bossenbroek H, et al. A three-gene leukaemic stem cell signature score is robustly prognostic in chronic myelomonocytic leukaemia. British Journal of Haematology. 2023;201(2):302-7.
2. Hurtado AM, Luengo-Gil G, Chen-Liang TH, Amaral F, Batta K, Palomo L, et al. Transcriptomic rationale for synthetic lethality-targeting ERCC1 and CDKN1A in chronic myelomonocytic leukaemia. Br J Haematol. 2018;182(3):373-83.
3. Khoury JD, Solary E, Abla O, Akkari Y, Alaggio R, Apperley JF, et al. The 5th edition of the World Health Organization Classification of Haematolymphoid Tumours: Myeloid and Histiocytic/Dendritic Neoplasms. Leukemia. 2022;36(7):1703-19.
4. Savona MR, Malcovati L, Komrokji R, Tiu RV, Mughal TI, Orazi A, et al. An international consortium proposal of uniform response criteria for myelodysplastic/myeloproliferative neoplasms (MDS/MPN) in adults. Blood. 2015;125(12):1857-65.
5. Wang Y-H, Lin C-C, Gurashi K, Wingelhofer B, Amaral FMR, Yao C-Y, et al. Higher MDMX expression was associated with hypomethylating agent resistance and inferior survival in MDS patients, inferring it a potential therapeutic target. Leukemia. 2023;37(12):2507-11.
6. Wiseman DH, Williams EL, Wilks DP, Sun Leong H, Somerville TDD, Dennis MW, et al. Frequent reconstitution of IDH2R140Q mutant clonal multilineage hematopoiesis following chemotherapy for acute myeloid leukemia. Leukemia. 2016;30(9):1946-50.
7. Wang Y-H, Hou H-A, Lin C-C, Kuo Y-Y, Yao C-Y, Hsu C-L, et al. A CIBERSORTx-based immune cell scoring system could independently predict the prognosis of patients with myelodysplastic syndromes. Blood Advances. 2021;5(22):4535-48.
8. Martin M. Cutadapt removes adapter sequences from high-throughput sequencing reads. 2011. 2011;17(1):3.
9. Dobin A, Davis CA, Schlesinger F, Drenkow J, Zaleski C, Jha S, et al. STAR: ultrafast universal RNA-seq aligner. Bioinformatics. 2013;29(1):15-21.
10. Love MI, Huber W, Anders S. Moderated estimation of fold change and dispersion for RNA-seq data with DESeq2. Genome Biol. 2014;15(12):550.
11. Unnikrishnan A, Papaemmanuil E, Beck D, Deshpande NP, Verma A, Kumari A, et al. Integrative Genomics Identifies the Molecular Basis of Resistance to Azacitidine Therapy in Myelodysplastic Syndromes. Cell Rep. 2017;20(3):572-85.
12. Darbaniyan F, Zheng H, Kanagal-Shamanna R, Lockyer P, Montalban-Bravo G, Estecio M, et al. Transcriptomic Signatures of Hypomethylating Agent Failure in Myelodysplastic Syndromes and Chronic Myelomonocytic Leukemia. Exp Hematol. 2022;115:44-53.
13. Meldi K, Qin T, Buchi F, Droin N, Sotzen J, Micol JB, et al. Specific molecular signatures predict decitabine response in chronic myelomonocytic leukemia. J Clin Invest. 2015;125(5):1857-72.

14. Zhang H, Nakauchi Y, Köhnke T, Stafford M, Bottomly D, Thomas R, et al. Integrated analysis of patient samples identifies biomarkers for venetoclax efficacy and combination strategies in acute myeloid leukemia. Nat Cancer. 2020;1(8):826-39.
15. Xu H, Muise ES, Javaid S, Chen L, Cristescu R, Mansueto MS, et al. Identification of predictive genetic signatures of Cytarabine responsiveness using a 3D acute myeloid leukaemia model. J Cell Mol Med. 2019;23(10):7063-77.
16. Williams MS, Amaral FM, Simeoni F, Somervaille TC. A stress-responsive enhancer induces dynamic drug resistance in acute myeloid leukemia. J Clin Invest. 2020;130(3):1217-32.
17. Gentles AJ, Plevritis SK, Majeti R, Alizadeh AA. Association of a leukemic stem cell gene expression signature with clinical outcomes in acute myeloid leukemia. Jama. 2010;304(24):2706-15.
18. Ng SW, Mitchell A, Kennedy JA, Chen WC, McLeod J, Ibrahimova N, et al. A 17-gene stemness score for rapid determination of risk in acute leukaemia. Nature. 2016;540(7633):433-7.
19. Eppert K, Takenaka K, Lechman ER, Waldron L, Nilsson B, van Galen P, et al. Stem cell gene expression programs influence clinical outcome in human leukemia. Nat Med. 2011;17(9):1086-93.
20. Ivanova NB, Dimos JT, Schaniel C, Hackney JA, Moore KA, Lemischka IR. A stem cell molecular signature. Science. 2002;298(5593):601-4.
21. Subramanian A, Tamayo P, Mootha VK, Mukherjee S, Ebert BL, Gillette MA, et al. Gene set enrichment analysis: A knowledge-based approach for interpreting genome-wide expression profiles. Proceedings of the National Academy of Sciences. 2005;102(43):15545-50.
22. Rodriguez-Meira A, Norfo R, Wen S, Chédeville AL, Rahman H, O'Sullivan J, et al. Single-cell multi-omics identifies chronic inflammation as a driver of TP53-mutant leukemic evolution. Nature Genetics. 2023;55(9):1531-41.
23. Aran D, Hu Z, Butte AJ. xCell: digitally portraying the tissue cellular heterogeneity landscape. Genome Biology. 2017;18(1):220.

Supplementary figures.

Supplementary Figure 1. *TP53* expression of acute myeloid leukemia (AML) and myelodysplastic syndromes (MDS) samples and normal cells.

Dot plots illustrating *TP53* expression in AML and MDS samples with selected karyotypes compared to normal cells. Data were extracted from BloodSpot (https://www.fobinf.com/). Human normal hematopoietic cells are from GSE42519; human AML/MDS cells are from GSE13159, GSE15434, GSE61804, GSE14468, and The Cancer Genome Atlas (TCGA). Abbreviations are defined in the table embedded below.

Supplementary Figure 2. *TP53* expression of CMML samples and healthy controls (HC).

Box plots displaying *TP53* expression of HC vs CMML in the three cohorts (A), *TP53* expression of CMML patients across different cohorts (left) and of HC among the three cohorts (right) (B). MNC: mononuclear cells. ****$P \leq 0.0001$, ***$P \leq 0.001$, **$P \leq 0.01$, *$P \leq 0.05$. *P*-values were computed using Mann–Whitney test.

Supplementary Figure 3. Comparison of *MDMX* and *MDM2* expression among CMML patients from the discovery and validation cohorts.

Box plots displaying expression of *MDMX* and *MDM2* in CMML patients with lower and higher *TP53* expression in each cohort. MNC: mononuclear cells. $^{**}P \leq 0.01$, $^{*}P \leq 0.05$. *P*-values were computed using Mann–Whitney test.

Supplementary Figure 4. Kaplan-Meier survival curves stratified by *TP53 expression* and existing risk stratification systems: CPSS.

Low *TP53* expression consistently conferred inferior acute myeloid leukemia-free survival and overall survival in patients across CMML-specific prognostic scoring system lower-risk (low and intermediate-1) (A-B) and higher-risk (intermediate-2 and high) groups (C-D).

Supplementary Figure 5. Kaplan-Meier survival curves stratified by *TP53 expression* and existing risk stratification systems: CPSS-Molecular.

Low *TP53* expression consistently conferred inferior acute myeloid leukemia-free survival and overall survival in patients across CMML-specific prognostic scoring system-molecular lower-risk (low and intermediate-1) (A-B) and higher-risk (intermediate-2 and high) groups (C-D).

Supplementary Figure 6. Prognostic implication of *TP53* expression was validated in two independent cohorts.

(A-B) Low *TP53* expression conferred significantly inferior acute myeloid leukemia-free survival (LFS) (A) and overall survival (OS) (B) in the UK cohort. (C-D) Patients with lower *TP53* expression consistently had inferior LFS (C) and OS (D) in the Spain cohort.

Supplementary Figure 7. Time-dependent ROC curves analysis revealed that incorporating *TP53* expression were complimentary to current prognostic systems in the UK cohort (A) and the Spain cohort (B).

Supplementary Figure 8. Representative bone marrow sections stained by immunohistochemistry (IHC) for p53 expression from CMML patients in the UK cohort

(A-B) Representative bone marrow sections with lower (A) and higher (B) p53 IHC expression from CMML patients in the UK cohort. Nuclei with a clear brown color, regardless of staining intensity, were considered p53 positive. The yellow area indicates p53 positive nuclei after filtering out background noise. Left to right: low-power field, high-power field, and high-power field with filtering.

Supplementary Figure 9. Biological implications of lower *TP53* expression in drug resistance.

Box plots displaying resistance signatures derived from single-sample GSEA for hypomethylating agents (HMA), venetoclax, cytarabine, and daunorubicin in patients with lower and higher *TP53* expression across the discovery cohort (A) and the Spain validation cohort (B). $^{**}P \leq 0.01$, $^{*}P \leq 0.05$. *P*-values were computed using Mann–Whitney test.

Supplementary Figure 10. Functional analysis of *TP53*^{low} vs *TP53*^{high} and healthy controls. Box plots displaying scores of leukemic stem cell (LSC), hematopoietic stem cell (HSC), and cell cycle of patients with *TP53*^{high} and *TP53*^{low} expression and healthy controls (HC) across the discovery cohort (A) and the Spain validation cohort (B). $^{****}P \leq 0.0001$, $^{**}P \leq 0.01$, $^{*}P \leq 0.05$. *P*-values were computed using Kruskal–Wallis test.

Supplementary Figure 11. Gene set enrichment analysis (GSEA) comparing bone marrow transcriptome of CMML patients and healthy controls across three cohorts. (A-F) Venn diagrams illustrating overlapped results across cohorts (left of each panel) and pathways enriched in at least 2 cohorts. (A-B) Pathways enrichment in CMML patients with lower vs higher *TP53* expression. (C-D) Pathway enrichment in CMML patients with the lowest 25% vs highest 25% *TP53* expression. (E-F) Pathway enrichment in CMML patients with the lowest 25% *TP53* expression vs healthy controls. RED BOLD FONT RB-E2F Related

Pathways; GREEN BOLD FONT Cell Cycle/Cell Division Pathways; BLUE BOLD FONT Inflammation Related Pathways.

Supplementary Figure 12. Therapeutic implications of *TP53* expression in CMML

(A) 3D synergy plots using the zero interaction potency (ZIP) model to calculate the synergy scores of the NSC-207895 and azacitidine combination at various concentrations, treating bone marrow mononuclear cells (n=3; mean ± SEM) from CMML patients for 72 hours. The presence of synergy was determined utilizing the SynergyFinder computational package and the ZIP synergy index where red denotes synergism and green denotes antagonism. A positive synergy score is the percent more cell death than expected. (B) Dot plot comparing synergy scores between patients with lower and higher *TP53* expression. *P*-values were computed using Mann–Whitney test.

Supplementary Figure 1.

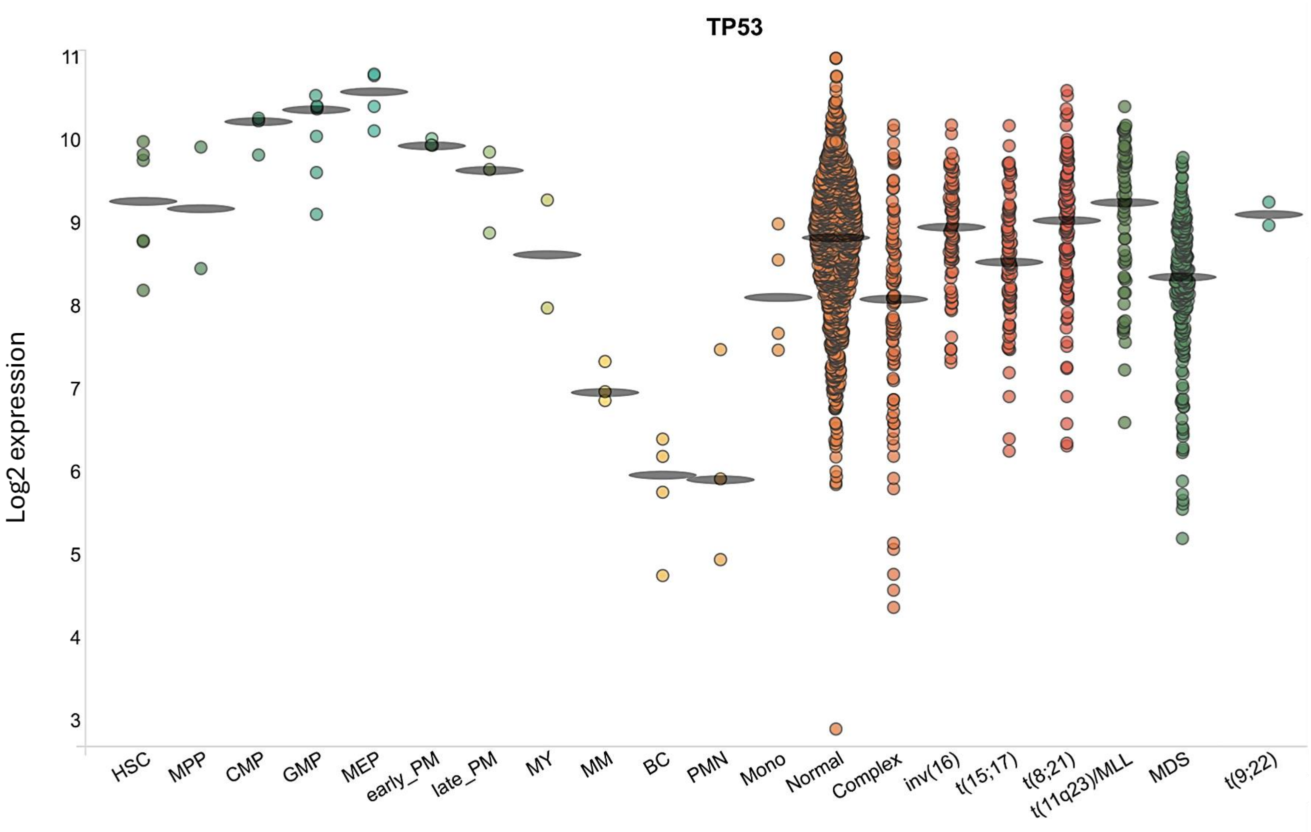

Short	Abbreviation
HSC	Haematopoietic stem cell
MPP	Multipotential progenitors
CMP	Common myeloid progenitor cell
GMP	Granulocyte monocyte progenitors
MEP	Megakaryocyte-erythroid progenitor cell
early_PM	Early Promyelocyte
Late_PM	Late Promyelocyte
BC	Band cell
MM	Metamyelocytes
MY	Myelocyte
Mono	Monocytes
PMN	Polymorphonuclear cells
Normal	AML with Normal karyotype
Complex	AML with Complex karyotype
inv(16)	AML with inv(16)
t(15;17)	AML with t(15;17)
t(8;21)	AML with t(8;21)
t(11q23)/MLL	AML with t(11q23)/MLL
MDS	Myelodysplastic sundromes
t(9;22)	AML with t(9;22)

Supplementary Figure 2.

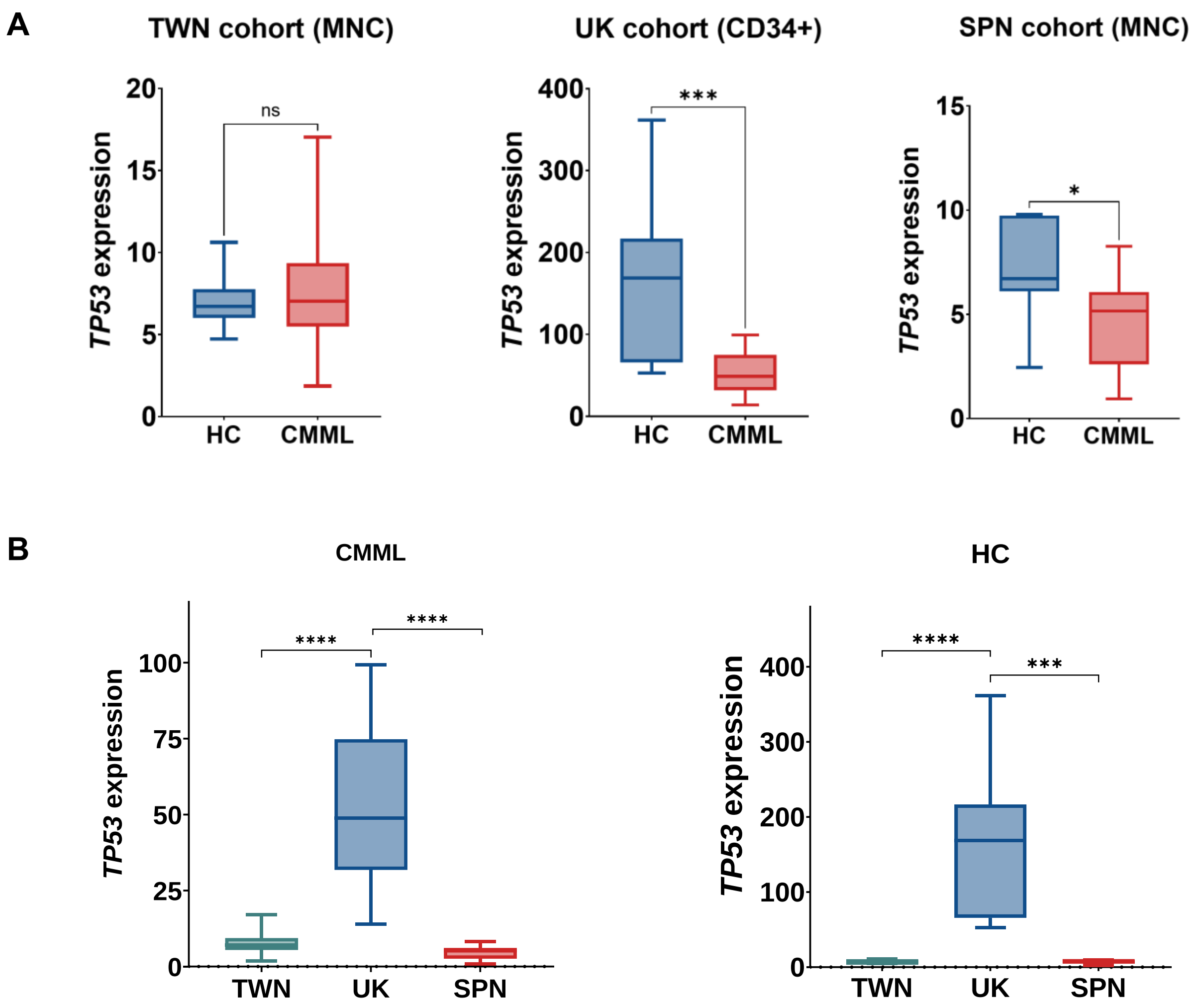

Supplementary Figure 3.

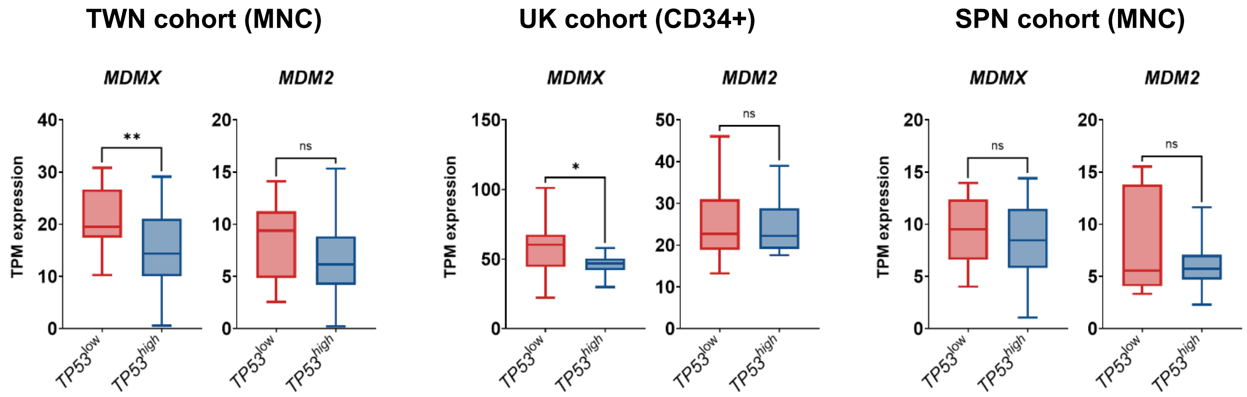

Supplementary Figure 4.

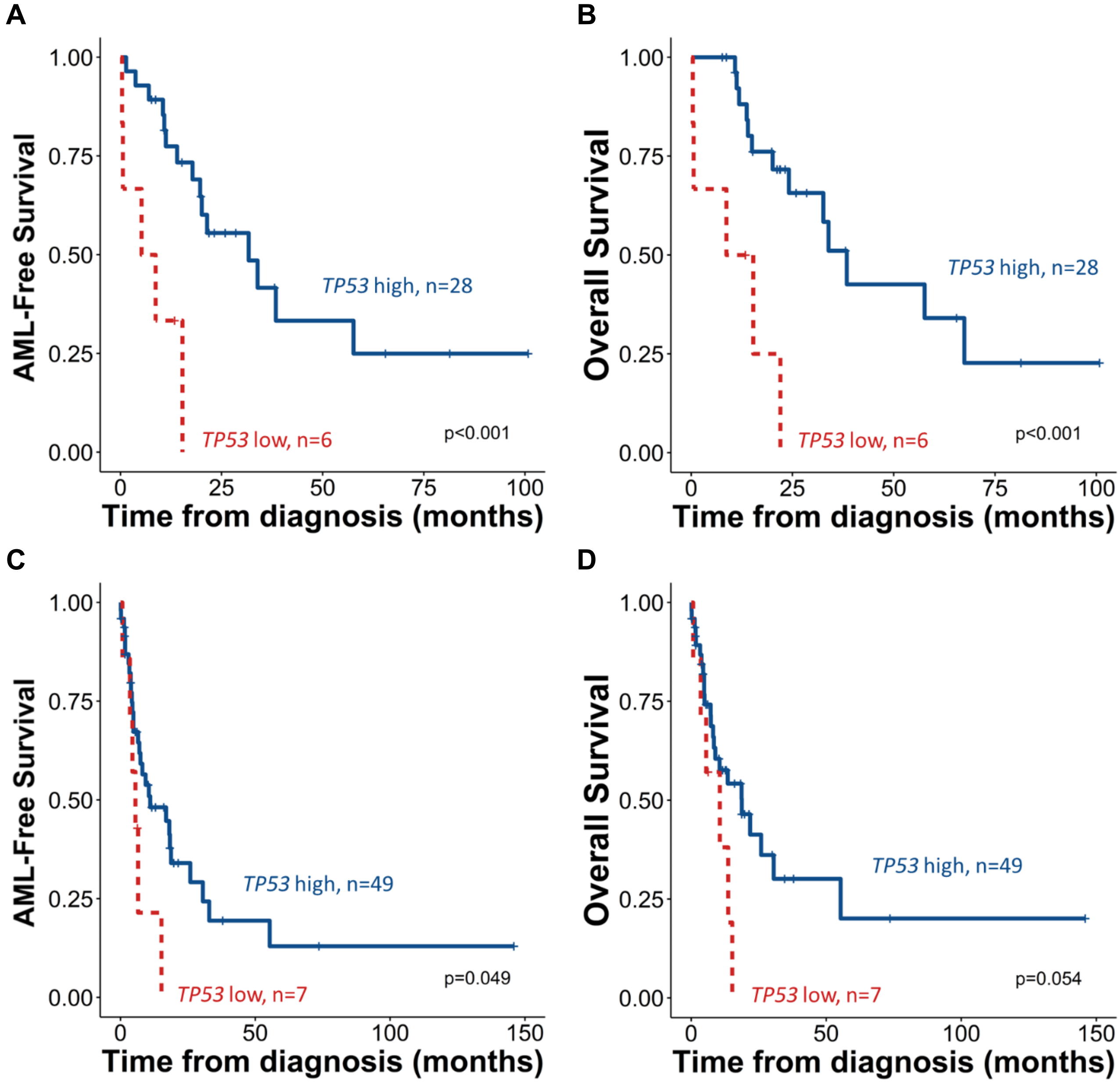

Supplementary Figure 5.

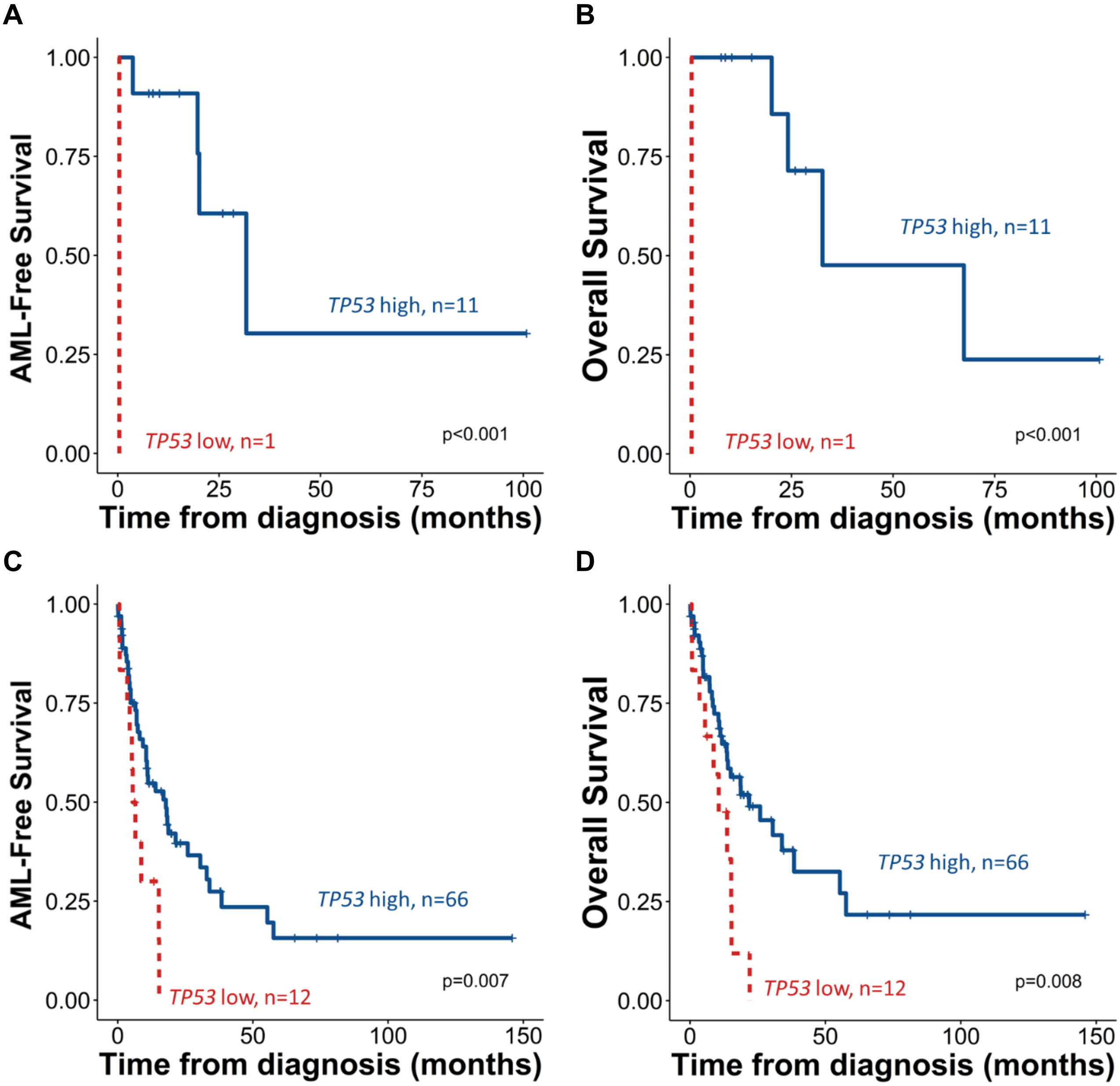

Supplementary Figure 6.

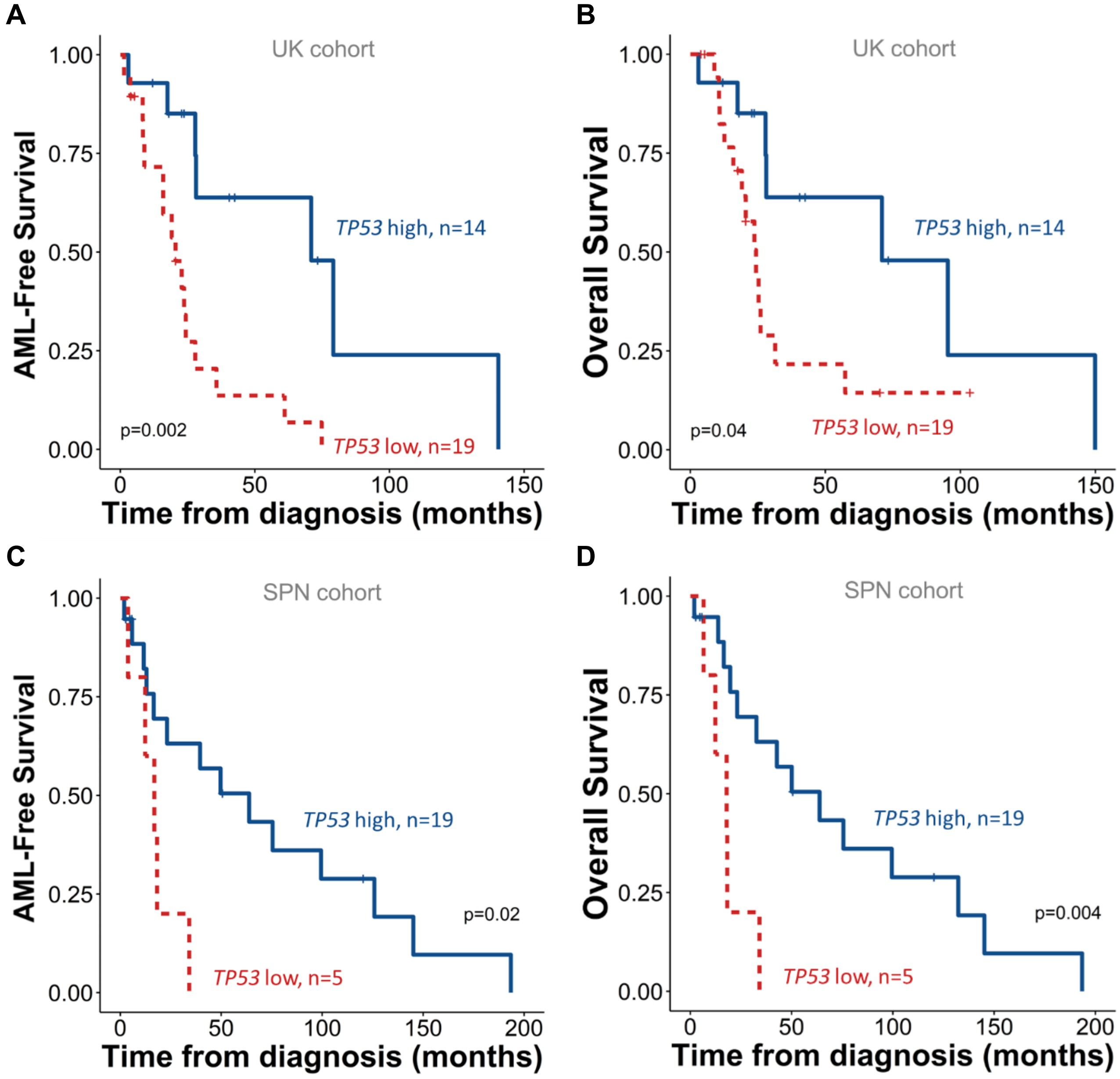

Supplementary Figure 7.

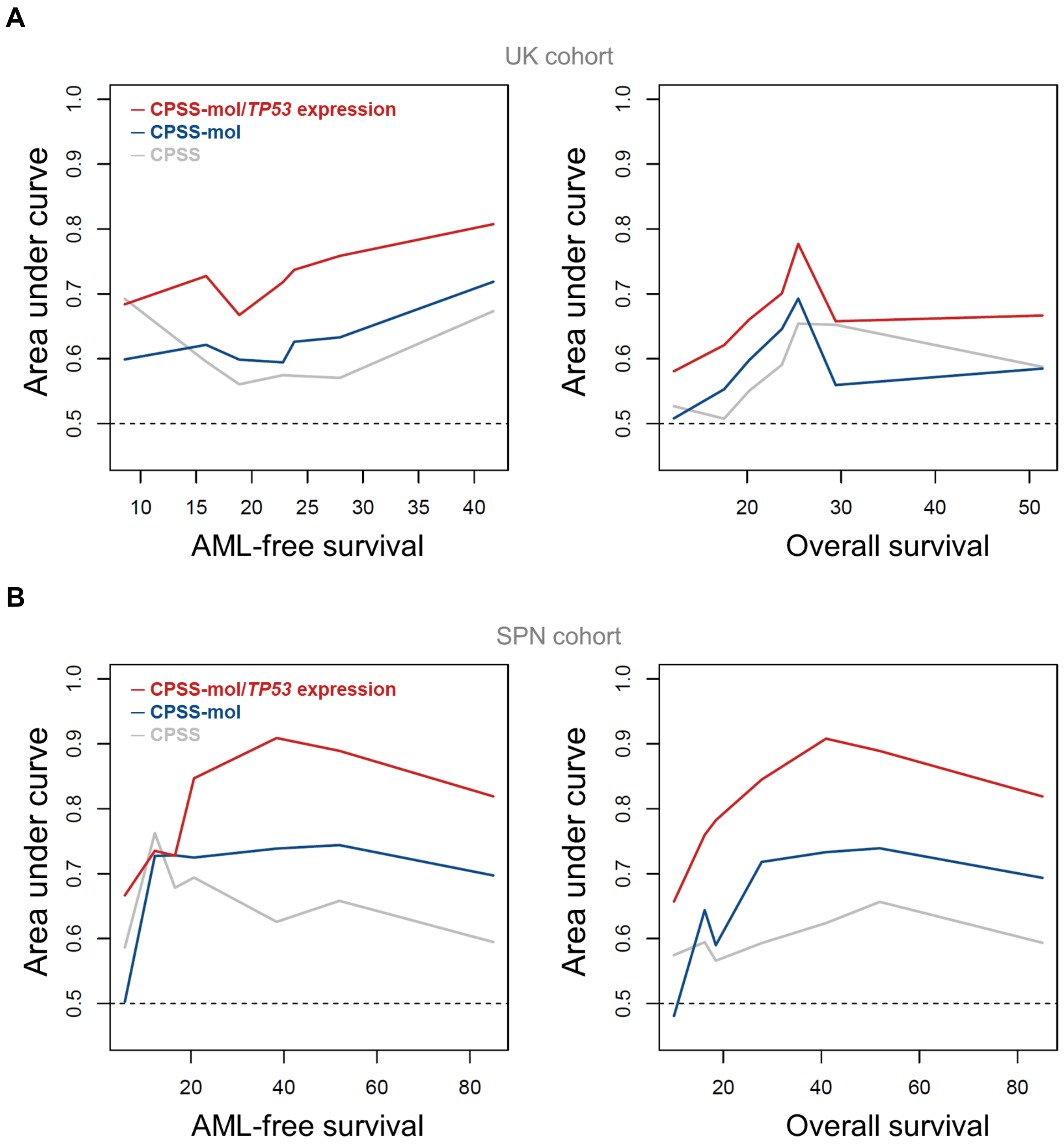

Supplementary Figure 8.

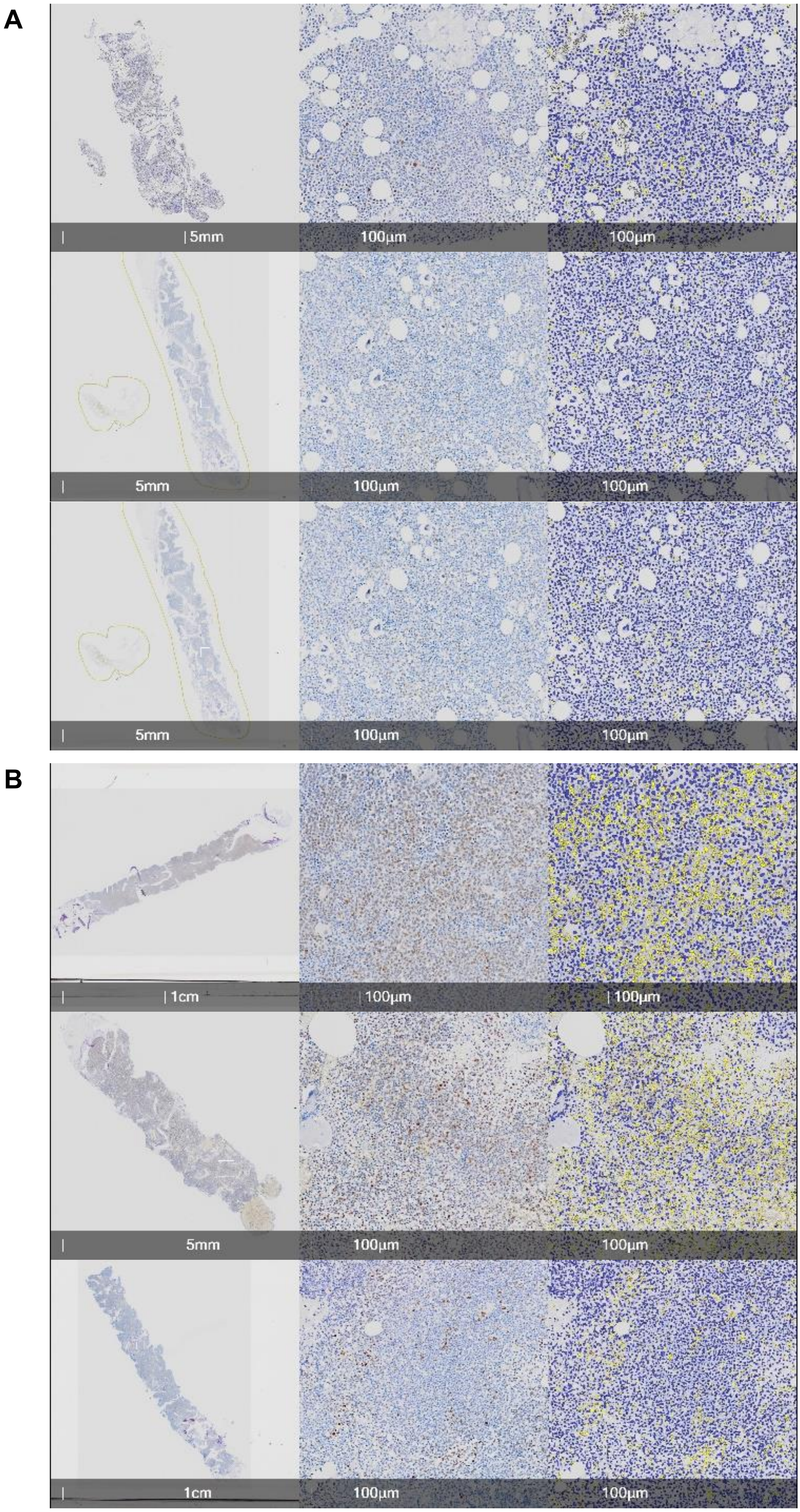

Supplementary Figure 9.

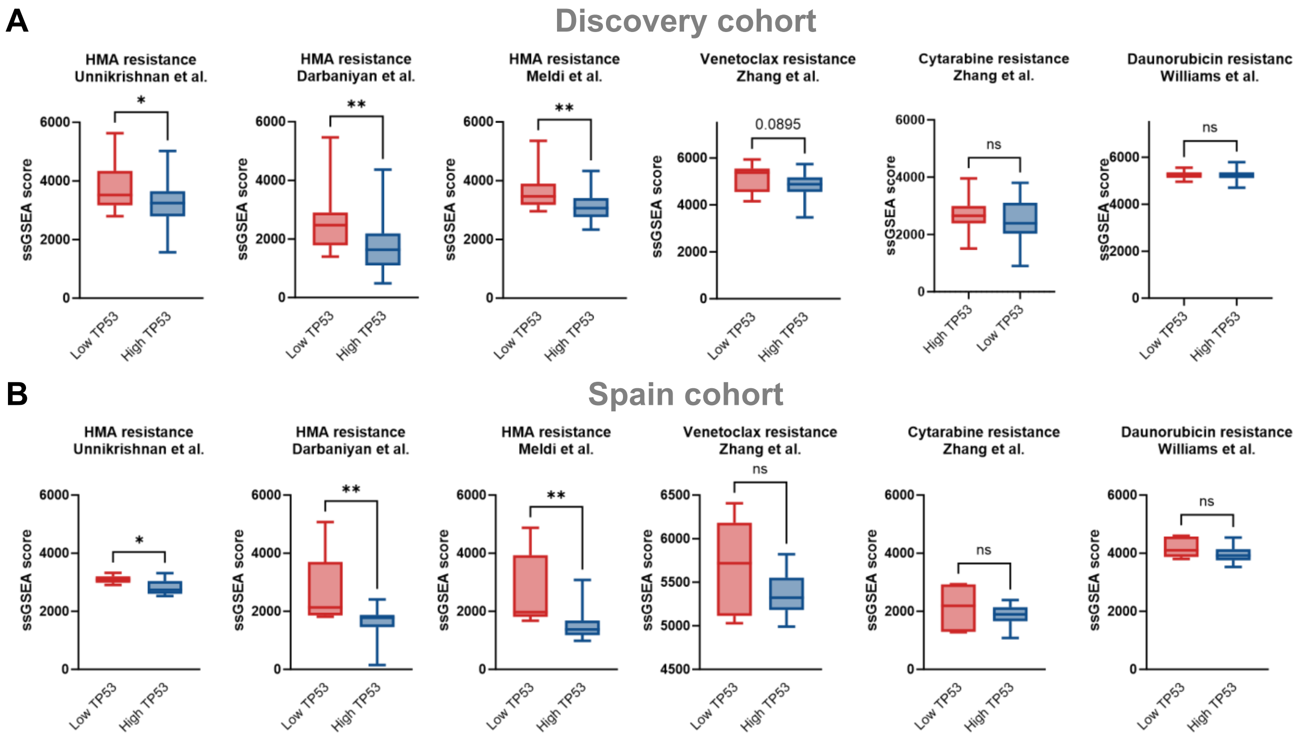

Supplementary Figure 10.

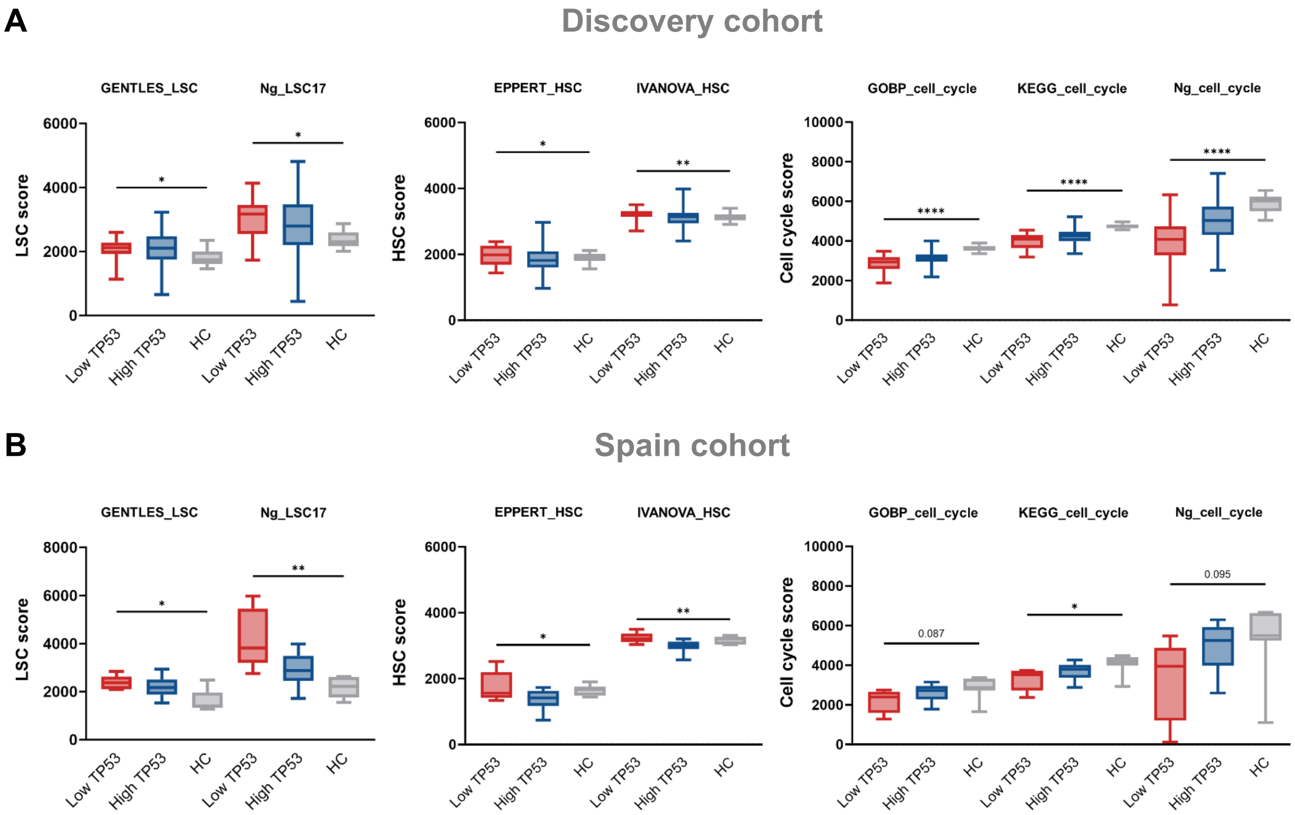

Supplementary Figure 11.

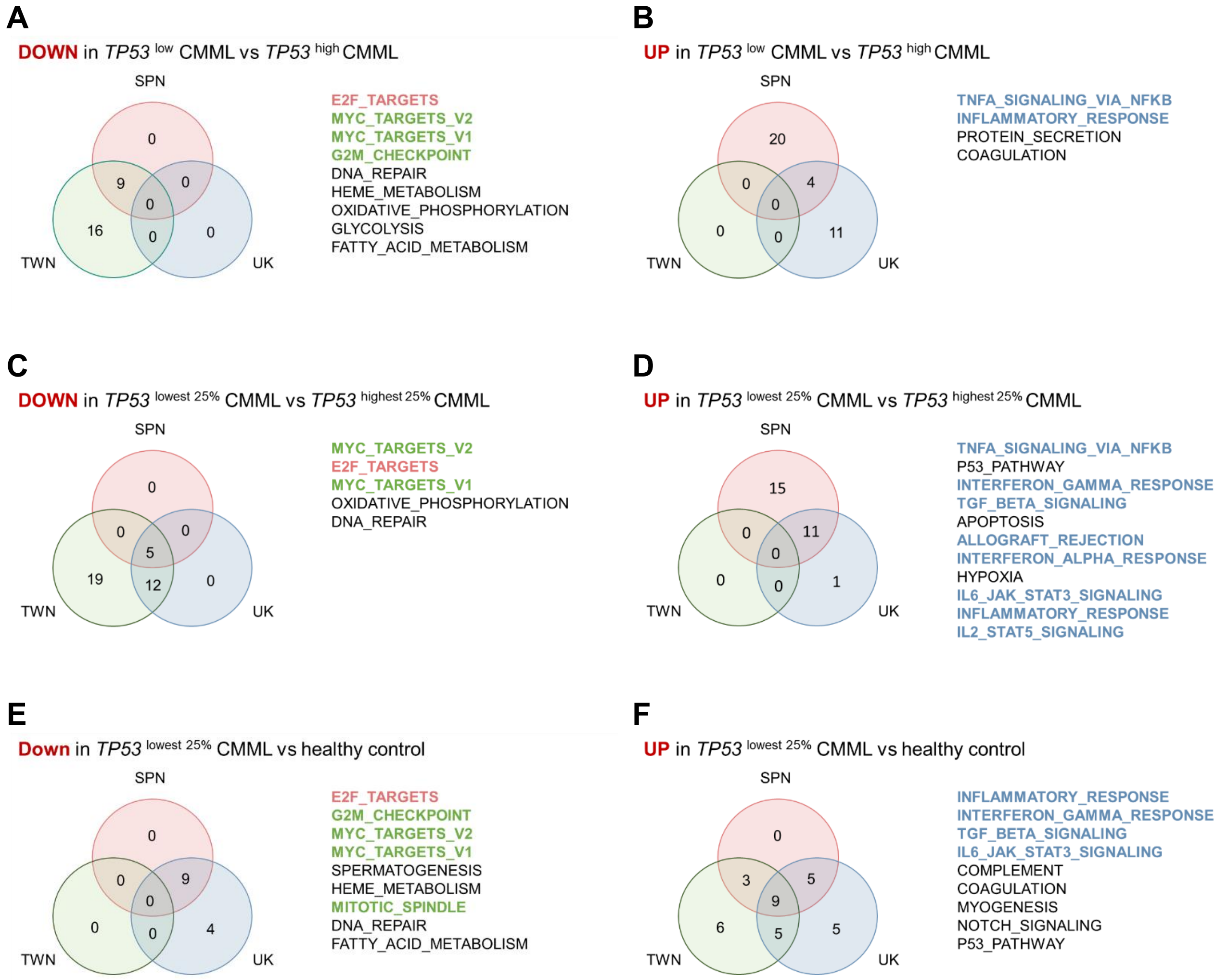

Supplementary Figure 12.

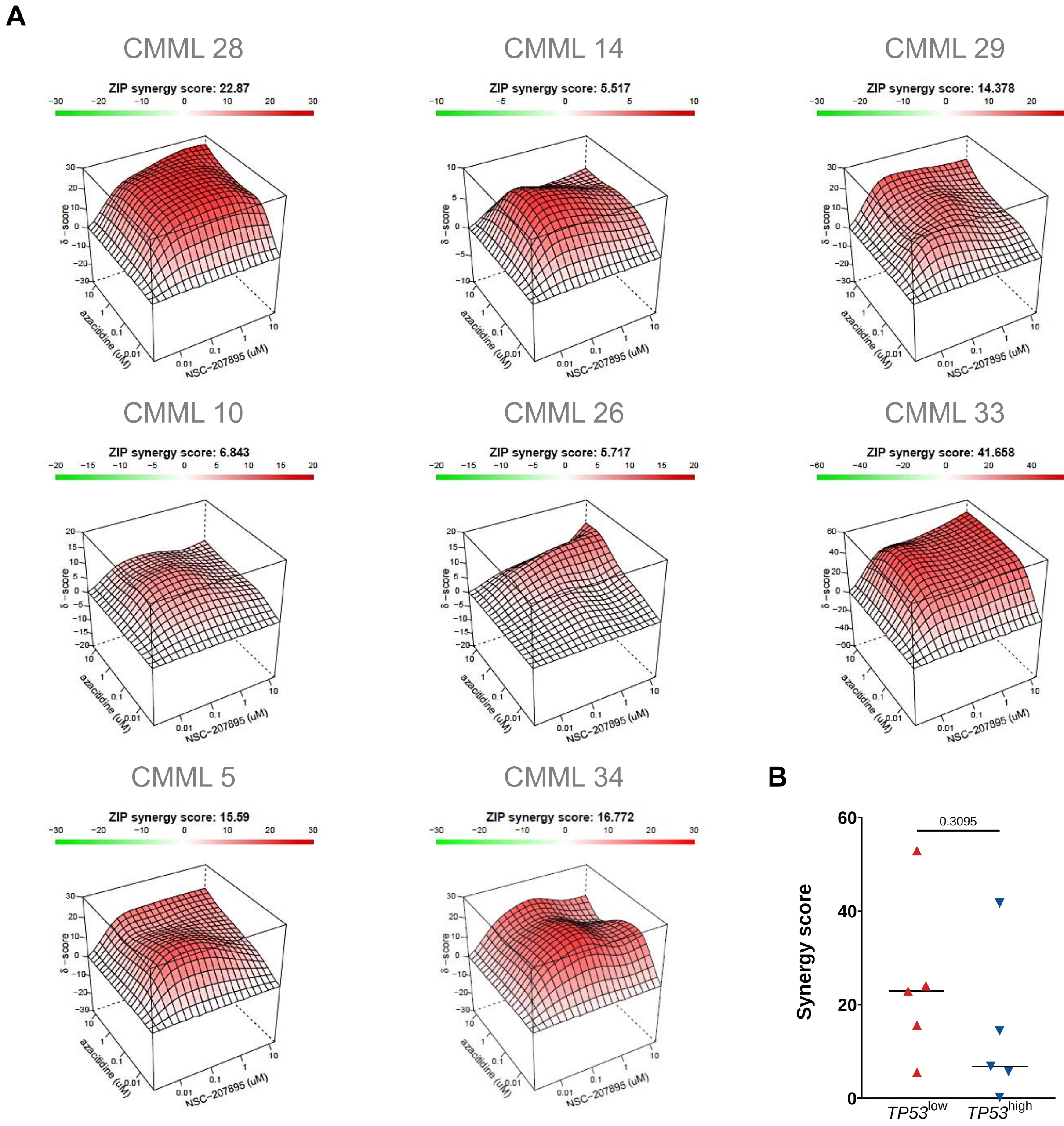

Supplement: Supplementary file 1 — Supplementary material [file 41408_2024_1087_MOESM1_ESM.pdf]
